# Maternal Hemoglobin Levels during Pregnancy and their Association with Birth Weight of Neonates

**Published:** 2015-12-10

**Authors:** F Moghaddam Tabrizi, S Barjasteh

**Affiliations:** 1 Assistant Professor in Nursing and Midwifery Department, Reproductive Health Research Center , Urmia University of Medical Sciences, Urmia, Iran.; 2 Midwifery Masters Degree Student, Student Research Center, Urmia University of Medical Sciences, Urmia, Iran

**Keywords:** Hemoglobin Level, Birth Weight, Pregnancy

## Abstract

**Back ground:**

Anemia in pregnancy is associated with increased rates of maternal and perinatal mortality, premature delivery, low birth weight, and other adverse outcomes

**Materials and Methods:**

A prospective study was conducted on 1405 Iranian pregnant women who delivered during 2015. Blood was collected from all the subjects to measure the hemoglobin (Hb) during 16-19 weeks, 22-24 weeks, and 34-36 weeks of gestation. According to the level of hemoglobin, it is divided into 4 groups. Group 1; Hb > 10.1 gm/100ml (control group), Group 2; Hb= 8.1-10 gm/100ml (mild anemia) Group 3; Hb= 6.5-8 gm/100ml (moderate anemia) Group 4; Hb <6.5 gm/100ml (severe anemia). After delivery, the neonates were weighted within 24 hours after birth. Maternal hemoglobin and birth weights were compared.

**Results:**

The anemia prevalence was 20.2% (Hb<10g/dl). Out of them, 16.2 % hadmoderate anemia (Hb=6.5-8 g/dl) and 83.8% had mild anemia (Hb=8.1-10 g/dl). Severe anemia did not detect in pregnant women. The hemoglobin levels in non anemic group showed a drop in the second trimester. Pregnant women with hemoglobin less than 10 g/dl, considered as anemic gave birth to neonates with birth weight of 2.6kg, while pregnant women with higher hemoglobin level (>10 g/dl), considered as normal gave birth to heavier and normal babies (3.3 kg). The severity of anemia during three trimesters was closely associated with birth weight of newborns.

**Conclusion:**

The low hemoglobin values during three trimesters of pregnancy were associated with low birth weight in Iran. The anemia can lead to intra uterine growth retardation.

## Introduction

Anemia is a major health problem that affects 25% to 50% of the population of the world and approximately 50% of pregnant women (1). Anemia in pregnancy is associated with increased rates of maternal and perinatal mortality, premature delivery, low birth weight, and other adverse outcomes (2). Barooti in a systematic review illustrated that the frequency of anemia in Iran is 4.8-17.5 percent (3).

During pregnancy, anemia increased more than fourfold from the first to third trimester (4). It is a well established fact that there is a physiological drop in hemoglobin (Hb) in the mid trimester (5). This physiological drop is attributed to increase of plasma volume and hence decrease of blood viscosity (6) lead to better circulation in placenta (7).

Research has shown that Hb and Hematocrit (Hct) concentrations typically decrease during the first trimester and reach the lowest levels at the end of second trimester and increase again during the third trimester of pregnancy (9). According to the classification of World Health Organization (WHO), pregnant women with hemoglobin levels less than 11.0 g/dl in the first and third trimesters and less than 10.5 g/dl in the second trimester are considered anemic (Table I) (11). Anemia in pregnant women detrimental to fetal growth and pregnancy outcomes (12, 13). Low birth weight and preterm delivery have been persistently linked to anemia in pregnancy (14-17). Yi et al., revealed that anemia, but not hemoglobin concentration, before pregnancy was associated with an elevated risk of preterm delivery (18). Kozuki et al reported that moderate to severe, but not mild, maternal anemia appears to have an association intra uterian growth retardation (19). An improvement in prenatal mean hemoglobin concentration linearly increased birth weight (14). In another study, low birth weight and small for gestational age increased with severity of anemia in Korean women (18).Haggaz et al. in a meta-analysis showed that anemia during early pregnancy, but not during late pregnancy was associated with slightly increased risk of preterm delivery and low birth weight(20), Whereas Ahankari et al., in systematic review study found that anemia in the first and third trimesters was associated with increased risk of low birth weight and they emphasized that hemoglobin needs to be routinely investigated during pregnancy, and women with low level of hemoglobin should be treated to minimize harmful impact on neonatal health (1). Based on literature review, the assessment of hemoglobin level in which trimester should be taken as standard is still not clear. Thus, it is important to understand the most vulnerable time for the fetus due to anemia in pregnancy. The present study was designed to observe the effect of hemoglobin levels during various trimesters of pregnancy for batter pregnancy outcome and fetal growth.

## Materials and Methods:

This study was conducted in health care centers which are located in Urmia. At the end of pregnancy, they referred to hospitals for delivery. A total number of 1405 healthy women, aged between 17 - 45 years, after pregnancy confirmation and undergoing prenatal care during the three trimesters of pregnancy, were registered as subjects for the present study. The study was approved by the Human Ethical Committee of Urmia Medical Science University. Written consent letters were obtained from all subjects. The inclusion criteria of the current study are the pregnant women without diabetes mellitus, cardio vascular disease (CVD), parathyroid, thyroid, bone, multiple pregnancies, mothers with placenta previa and placenta abruptia. Blood was collected from all subjects to measure the hemoglobin concentration during 16-19 weeks, 22-24 weeks and 34-36 weeks of gestation. The selected method for hemoglobin assessment was Cyanomethemoglobin (W.H.O/ UNICEF/ UNO, 1998). According to the level of hemoglobin. patients were divided into 4 groups; Group 1; Hb > 10.1 gm/100ml (control group), Group 2; Hb= 8.1-10 gm/100ml (mild anemia) Group 3; Hb=. 6.5-8 gm/100ml (moderate anemia) Group 4; Hb <6.5 gm/100ml (severe anemia).

The weight of patients were measured using standard procedure within 24 hours after birth (21). A baby beam balance with accuracy of 50 g was employed for weighing the infants. Infants were weighed with minimum clothing while the child was restful. Due to normal distribution of all data, analysis of variance (ANOVA) was used for determination of differences three groups followed by post hoc test.

## Results

In the present study, 1405 pregnant women were selected and changes in hemoglobin concentration during 3 trimesters and its relation to birth weight of neonates were studied. Mothers' demographic profile has been shown in Table 2. The mean age of pregnant women was 26.1±5.8 years old and ranged from 18 to 40 years old. Majority (41%) of pregnant women was in the age group of 26-36 years old. Hemoglobin content during the three trimesters of pregnancy is presented in Table 3. As Table 3 illustrates the anemia prevalence was 20.2% (Hg<11g/dl). Out of them, 16.2 % had moderate anemia (Hg=7-8.9 g/dl) and 83.8% had mild anemia (Hg=9-10.9 g/dl). Severe anemia did not detected in pregnant women in the present study. It is clear from Table 3 that there was significant difference in the mean hemoglobin level in non anemic women during the three trimesters. The mean hemoglobin level during the second trimester showed significantly drop (p<0.05) in second trimester of pregnancy. The levels of hemoglobin in the present study were categorized into different levels on the basis of WHO classification. According to the classification of the World Health Organization, pregnant women with hemoglobin levels less than 10 g/dl on the first and the third trimesters were categorized as anemic women. Women with anemia during the third trimester of pregnancy and who had hemoglobin levels 8.1-10g/dl, 6.5-8 g/dl and <6.5g/dl were classified as having mild, moderate and severe anemia respectively (WHO/UNICEF/UNO.IDA, 1998). As shown in Table 4, pregnant women with hemoglobin less than 10 g/dl, considered as anemic gave birth to neonates with birth weight of 2.6kg, while pregnant women with higher hemoglobin level (>10 g/dl), considered as normal, gave birth to heavier and normal babies (3.3 kg). These findings showed that the hemoglobin level of pregnant women increased and the birth weight of the neonates also increased. Different superscripts indicate significant difference at 5 % level as shown by Post hoc Bonferroni test.

**Table I:** Hb adjustments for an unknown trimester by WHO (10).

	Hemoglobin (g/dl)
First	-1.0g/dl
Second	-1.5g/dl
Third	-1.0g/dl
Unknown	-1.0g/dl

**Table II:** Demographic Information on Pregnant Women (n=1405)

	Category	N	Percent
Age Group(years)	<20	253	18
	20-26	506	36
	26-36	576	41
	>36	70	5
Mean Age(years)	26.1±5.8		
Education Level			
	≤Secondary	253	18
	High School& Diploma	674	48
	College Graduated	478	34
Financial Status			
	No money problem	281	20
	Fair	717	51
	Not enough	407	29

**Table III:** Comparison of changes in hemoglobin concentration (Mean ± SD) during three trimesters of pregnancy

	Hemoglobin concentration (g/dl)					
	Non Anemic (79.8%)		Anemic (20.2%)			
Trimesters	n=1121(79.8%)	p value	Mild n=83 (83.8%)	P value	Moderate n=46(16.2%)	P value
First	10.65±1.90^a^	0.035	9.40±1.40^a^	0.082	8.33±1.08^a^	0.042
Second	9.395±1.10^b^		9.42±1.81^a^		8.38±0.99^a^	
Third	11.30±0.83^c^		8.99±0.80^a^		7.31±0.07^b^	

* Statistical Significant at P<0.05

**Table IV:** Classification of Anemia Based on Hemoglobin Assessment during Three Trimesters of Pregnancy (n=1405

Trimesters Hemoglobin	Classification of Anemia†	Levels of hemoglobin concentration (g/dl)	Birth Weight(g)
First	Moderate	6.5-8	2609±431^a^
	Mild	8.1-10	2701±512^b^
	Normal	>10.1	3216±724^c^
Second	Moderate	6.5-8	2615±611^a^
	Mild	8.1-10	2697±610^b^
	Normal	>10.1	3271±504^c^
Third	Moderate	6.5-8	2604±634^a^
	Mild	8.1-10	2737±701^b^
	Normal	>10.1	3301±623^c^

† According the classification of the world health organization (WHO/UNICEF/UNO.IDA, 1998).

* Statistical Significant at P<0.05.

## Discussion

This study demonstrated that the prevalence of anemia is 20.2% (n=1405) among Iranian pregnant women in Urmia. Other studies have shown a higher degree of anemia in pregnancy such as 87% in India (22) , 58.6% in China (23), 50% in South Asia (24), and 43% in Turkey(25) .

The low prevalence of anemia in the present study may be related to more frequent iron supplementation consumption. Since the women had more visits for prenatal care so in each visit they were encouraged to take their supplements. Therefore, it seems that iron deficiency anemia is relatively lower in this study in compared to other studies. Based on the results, the hemoglobin levels in the non anemic group showed a drop in second trimester. Again the hemoglobin level increased in third trimester and it was realized to be similar to the first trimester. 

These are comparable with the mean hemoglobin level reported in pregnancy in other studies (26-29), which they found a U-shaped curve of hemoglobin levels during pregnancy, with higher mean hemoglobin in early pregnancies (12-16 weeks) and in late pregnancies (≥37weeks) than in mid-pregnancy (28-33 weeks). The reduction observed in hemoglobin mean during the second trimester of pregnancy is related to the plasma expansion. The mid-trimester drop seen in non anemic mothers was not seen in anemic mothers. Similar results were found in other studies (22, 30). The mean birth weight of the babies in both groups of anemic and nonanemic mothers was in clinical normal range irrespective of the trimester. There were significant differences in birth weight values among three groups during three trimesters. The higher hemoglobin levels are associated with higher birth weight values. Babies born to the anemic mothers had lower birth weight compared to nonanemic mothers. Other studies (14, 28, 29, 32, 33) are in agreement with the current study and demonstrate indicated the importance of normal hemoglobin level on pregnancy outcome. There is a substantial amount of evidence showing that maternal iron deficiency anemia during pregnancy can be resulted in low birth weight (34). For example, Bodeau-Livinec et al., found women without anemia during the third trimester, in compared to women with severe anemia were at higher risk of low birth weight after adjustment for potential confounding factors (35). 

## Conclusion

This study showed that low prenatal hemoglobin status is associated with low birth weight in neonates.
